# The clinical value and usage of inflammatory and nutritional markers in survival prediction for gastric cancer patients with neoadjuvant chemotherapy and D2 lymphadenectomy

**DOI:** 10.1007/s10120-019-01027-6

**Published:** 2020-02-18

**Authors:** Ziyu Li, Shuangxi Li, Xiangji Ying, Lianhai Zhang, Fei Shan, Yongning Jia, Jiafu Ji

**Affiliations:** Key Laboratory of Carcinogenesis and Translational Research (Ministry of Education/Beijing), Department of Gastrointestinal Surgery, Peking University Cancer Hospital & Institute, Haidian District, 100142 Beijing China

**Keywords:** Gastric cancer, Neoadjuvant treatment, Anemia, Inflammation, Nutrition

## Abstract

**Background:**

The clinical values of inflammatory and nutritional markers remained unclear for gastric cancer with neoadjuvant chemotherapy (NACT).

**Methods:**

The inflammatory, nutritional markers and their changes were analyzed for locally advanced gastric cancer with NACT. The predictive value was evaluated by the Cox proportional hazards regressions under three hypothesized scenarios. The nomograms including independent prognostic factors were plotted for survival prediction.

**Results:**

A total of 225 patients were included in the study. The neutrophil-to-lymphocyte ratio (NLR), platelet-to-lymphocyte ratio, lymphocyte-to-monocyte ratio (LMR), systemic immune-inflammation index, and hemoglobin (Hgb) were significantly reduced, and the body mass index was significantly increased after NACT (all *P* < 0.05). The pre-NACT NLR [hazard ratio (HR) = 1.176, *P* = 0.059] showed a trend to correlate with the overall survival (OS) when only pre-NACT markers available; The post-NACT Hgb (HR = 0.982, *P* = 0.015) was the independent prognostic factor when only post-NACT markers available; The post-NACT Hgb (HR = 0.984, *P* = 0.025) and the change value of LMR (HR = 1.183, *P* = 0.036) were the independent prognostic factors when both pre- and post-NACT markers available. The nomogram had a similar Harrell’s C-statistic compared to ypTNM stage (0.719 vs. 0.706).

**Conclusion:**

For locally advanced gastric cancer, the NACT could significantly decrease some inflammatory markers. The pre-NACT NLR, the post-NACT Hgb and the change value of LMR had some values in survival prediction combined with age, sex, tumor location and the clinical stages under different clinical scenarios. The elevated initial NLR, the preoperative anemia and the greater change value of LMR implied a poor prognosis.

**Electronic supplementary material:**

The online version of this article (10.1007/s10120-019-01027-6) contains supplementary material, which is available to authorized users.

## Introduction

Gastric cancer remains the fifth-most prevalent and the third leading cause of cancer death worldwide according to the latest epidemiologic data [1]. Although radical resection has always been the core method in curing locally advanced gastric cancer, perioperative/neoadjuvant chemotherapy (NACT) was verified its long-term survival advantage over surgery alone in several randomized clinical trials [2, 3], and thus was recommended as the preferred option for locally advanced stages according to some guidelines [4, 5]. Clinically, NACT brings several possible advantages, such as tumor down-staging, micro-metastasis control, and better tolerance compared to adjuvant chemotherapy.

The application of NACT in gastric cancer presented a demand for more accurate prediction on long-term survival for such patients. In the 8th edition of the American Joint Committee on Cancer (AJCC) staging system for gastric cancer, a novel ypTNM staging system has been proposed based on the United States National Cancer Database [6]. In addition, many efforts have been made to identify the prognostic markers for gastric cancer using other clinical, physiological or pathological parameters. Among them, the inflammatory markers [i.e., neutrophil-to-lymphocyte ratio (NLR), platelet-to-lymphocyte ratio (PLR), lymphocyte-to-monocyte ratio (LMR), systemic immune-inflammation index (SII), C-reactive protein–albumin ratio (CAR), modified Glasgow Prognostic Score (mGPS), Prognostic Index (PI), modified systemic inflammation score (mSIS)] and the nutritional markers [i.e., body mass index (BMI) and prognostic nutrition index (PNI)] were documented to have some values in prognosis prediction [7–14]. Summarily, the elevated inflammatory and the deteriorated nutritional status could predict worse outcomes for gastric cancer patients without any neoadjuvant treatments.

Considering the changes of patients’ hematological and biochemical status during NACT, another issue was raised as the impacts of NACT on the predicting values of the inflammatory and nutritional markers. This study was aimed to explore the issue for locally advanced gastric cancer with NACT. Subsequently, the nomograms were plotted based on the independent markers.

## Methods

### Patients

The study population was selected from a registered prospective observational cohort study (ClinicalTrials: NCT03493880). All the patients were treated in Peking University Cancer Hospital between February 2014 and June 2018. The inclusion criteria were as follows: (1) histologically confirmed gastric adenocarcinoma in biopsy; (2) clinical stage from II to IVA; (3) NACT performed for at least 2 cycles; (4) confirmed negative peritoneal cytology by diagnostic laparoscopy or abdominal paracentesis prior to NACT; and (5) gastrectomy performed with D2 lymphadenectomy. The exclusion criteria were as follows: (1) remnant gastric cancer; (2) neoadjuvant treatments other than chemotherapy; (3) emergency surgery performed in case of digestive bleeding or perforation; (4) combined with other malignancy; and (5) diagnosed with autoimmune disease or had a medication history of steroids longer than 1 month. The study was performed in accordance with the Declaration of Helsinki and approved by the Institutional Review Board of Peking University Cancer Hospital.

### Treatments and follow-ups

The NACT was performed for 2 cycles at least, and the regimens were all fluorouracil-based, the combined drugs included cisplatin, oxaliplatin, paclitaxel and docetaxel. The clinical assessments were carried out every 2–3 cycles, based on physical conditions, tumor markers and computed tomography. The adverse events were classified according to the Common Terminology Criteria for Adverse Events (CTCAE) [15]. For adverse events of 3 or 4 grades, necessary medical cares were conducted on patients, including full rest, supportive treatments, blood transfusion, and colony-stimulating factors.

The surgery was indicated when stable disease or progression disease acquired, or according to scheduled treatment plan, and was performed between the 4th and the 6th week after the completion of NACT. The surgical procedures were in accordance with Japanese Gastric Cancer Association (JGCA) treatment guideline [16], which involved the resection of at least two-thirds of stomach and peri-gastric lymphadenectomy with D2 extension. The postoperative complications were classified according to the Clavien–Dindo grading system [17]. Pathological response was evaluated by tumor regression grades (TRGs) [5]. The yield T stage, N stage, M stage, and TNM stage were classified according to the 8th AJCC Cancer Staging Manual for gastric cancer [6].

Adjuvant chemotherapy was routinely recommended for the patient whose Eastern Cooperative Oncology Group (ECOG) score was no more than 2. The regimens and cycles were determined by the oncologist based on the clinical and pathological responses. Follow-up was conducted every 3 months in the first 2 years, and every 6 months in the third to the fifth year after the discharge. The overall survival (OS) was defined as the time (in months) from the initial NACT to the date of death from any cause or last follow-up. The survival after surgery (post-NACT OS) was defined as the time from the date of surgery to the date of death or last follow-up.

### Data management

The clinical and pathological data, including demography, surgery, pathology, postoperative morbidity and mortality, were retrieved from a prospectively collected and maintained database of gastric cancer. The inflammatory and nutritional markers were calculated or rated from the peripheral blood tests in our laboratory department. The inflammatory markers included NLR, PLR, LMR, SII CAR, mGPS, PI and mSIS, and the nutritional markers included serum albumin (Alb), BMI and PNI. All these markers were calculated or rated as described previously [7–13] (Supplementary Table 1).

These markers were calculated or rated at two time-points, namely the timing of pre-neoadjuvant chemotherapy (pre-NACT) and the timing of post-neoadjuvant chemotherapy (post-NACT). The pre-NACT was defined as the time within 2 weeks before the initial NACT, and the post-NACT was defined as the time within 2 weeks before the surgery. The change in each marker was calculated by subtracting the pre-NACT value from the post-NACT value.

### Statistical analyses

Categorical data were presented as the numbers (percentage); continuous data were presented as the mean (± standard deviation) if normally distributed or as the median (interquartile range) if not normally distributed. Differences of categorical or continuous data between pre-NACT and post-NACT were analyzed by McNemar Chi square tests or paired *T* tests, respectively.

We hypothesized the following clinical scenarios to evaluate the clinical value of the markers for survival prediction, when only pre-NACT markers available, when only post-NACT markers available, and when both pre-NACT and post-NACT markers available. To adapt to the above scenarios, the Cox proportional hazards regressions were set accordingly.

When only pre-NACT markers were available, the age, sex, tumor location, and clinical stage were used as the adjustment to predict the OS. When only post-NACT markers were available, the age, sex, tumor location, post-NACT clinical stage was used as the adjustment to predict the post-NACT OS. When both pre-NACT and post-NACT markers were available, the age, sex, tumor location, and clinical stage were used as the adjustments to predict the post-NACT OS. The change values of the markers were analyzed as well. Under each hypothesized situation, the factors with a *P* value less than 0.1 in the univariate analyses were included in the Cox proportional hazards regression models (backward method) with the same adjustments.

The models with the independent prognostic factors were selected to plot the nomogram. The predictive values for survival were determined by the Harrell’s C-statistic (C-index). The goodness of fit was determined by the Akaike Information Criterion (AIC).

All analyses were performed with STATA^®^ (version 15.0). The nomogram was plotted with RStudio (version 1.1.463, with packages “Hmisc”, “lattice”, “Formula”, “ggplot2”, and “rms”). Statistical significance was declaimed with two-sided *p* < 0.05 for all tests.

## Results

### Clinical-pathological characteristics

Table 1 shows the clinical-pathological characteristics of the 225 patients included in the study. The median age was 60.0 (53.0 ~ 65.0) years. There were 172 (76.4%) male patients and 53 (23.6%) female patients. 153 (68.8%) patients were at clinical stage III, 59 (26.2%) at stage II, and 13 (5.8%) at stage IVA. As for NACT, 183 (81.3%) patients used platinum-based regimen, the chemotherapy was performed for 3.3 cycles at the average. The tumor located in lower, middle, upper and total part was 60.4%, 13.8%, 21.3%, and 4.4%, respectively. Accordingly, 127 (56.4%) patients were performed with distal gastrectomy, and 98 (43.6%) patients with total gastrectomy. The total postoperative morbidity was 24.4%, and the mortality was 0.4%. There were 4 patients who failed to initiate adjuvant chemotherapy due to poor physical conditions or postoperative mortality.

**Table 1:** Clinical–pathological data

Clinical data	Value	Pathological data	Value
Age (years)	60.0 (53.0 ~ 65.0)	Histologic type	
Sex		Ade	158 (70.2)
Male	172 (76.4)	Muc/sig	19 (8.4)
Female	53 (23.6)	Ade + (muc/sig)	47 (20.9)
Tumor location		Others	1 (0.4)
Upper	48 (21.3)	Lauren type	
Middle	31 (13.8)	Intestinal	90 (40.0)
Lower	136 (60.4)	Diffused	75 (33.3)
Total	10 (4.4)	Mixed	58 (25.8)
Clinical stage		N/A	2 (0.9)
II	59 (26.2)	TRG in primary lesion	
III	153 (68.8)	Grade 0^a^	23 (10.2)
IVA	13 (5.8)	Grade 1	37 (16.4)
Post-NACT clinical stage		Grade 2	90 (40.0)
I	11 (4.9)	Grade 3	75 (33.3)
II	71 (31.6)	ypT stage	
III	131 (58.2)	T0^a^	23 (10.2)
IVA	12 (5.3)	T1a–1b	32 (14.2)
Regimen for NACT		T2	36 (16.0)
Platinum-based	183 (81.3)	T3	81 (36.0)
Taxol-based	20 (8.9)	T4a–4b	53 (23.6)
Platinum–taxol-based	22 (9.8)	ypN stage	
Cycles for NACT	3.3(± 0.8)	N0	116 (51.6)
Surgery type		N1	50 (22.2)
Distal gastrectomy	127 (56.4)	N2	35 (15.6)
Total gastrectomy	98 (43.6)	N3a	17 (7.6)
Postoperative morbidity		N3b	7 (3.1)
Grade 0	170 (75.6)	Metastatic LN	0 (0 ~ 3)
Grade I–II	43 (19.1)	Total LN examined	33 (26 ~ 42)
Grade IIIa–IIIb	9 (4.0)	ypTNM stage	
Grade IV	2 (0.9)	PCR	22 (9.8)
Grade V	1 (0.4)	I^a^	55 (24.4)
Adjuvant chemotherapy		II	73 (32.4)
Yes	221 (98.2)	III	70 (31.1)
No	4 (1.8)	IV	5 (2.2)

Ade, adenocarcinoma; LN, lymph nodes; Muc, mucinous adenocarcinoma; NACT, neoadjuvant chemotherapy; PCR, pathological complete response; POD, postoperative days; Sig, signet-ring cell carcinoma; TRG, tumor regression grade

^a^One patient had ypT0N1 stage, and was assigned to ypTNM stage I

As for pathological features, there were 158 (70.2%) patients of adenocarcinoma, 47 (20.9%) patients of adenocarcinoma with signet ring cell carcinoma or mucinous adenocarcinoma. Regarding Lauren types, there were 90 (40.0%) patients of intestinal type, 75 (33.3%) patients of diffused type, and 58 (25.8%) patients of mixed type. Pathological complete response (PCR) was acquired in 22 (9.8%) patients, the TRG of 1, 2, and 3 grade was acquired in 37 (16.4%), 90 (40.0%), and 75 (33.3%) patients, respectively. The median number of metastatic and total lymph nodes was 0 (0 ~ 3) and 33 (26 ~ 42). The ypTNM stage of I, II, and III was diagnosed for 55 (24.4%), 73 (32.4%), and 70 (31.1%) patients (Table 1), respectively. There were 5 patients who were diagnosed as ypstage IV due to the reason of peritoneal cytology conversion from negative to positive after NACT, and no patient progressed to have distant metastasis preoperatively.

The median follow-up period was 29.8 (24.2 ~ 38.9) months, and the 3-year OS was 72.8% (65.3% ~ 78.9%), the 3-year post-NACT OS was 71.0% (62.9% ~ 77.6%). At the last follow-up (October 15th 2019), there were 51 deaths observed, including 49 gastric cancer-related death, 1 postoperative mortality, and 1 cerebral hemorrhage.

### Adverse events during NACT

Supplementary Table 2 shows the frequencies and percentages of the adverse events during NACT. The incidence of neutropenia, leucopenia, anemia, thrombocytopenia, lymphocytopenia, and hypoalbuminemia was 56.0%, 42.7%, 42.2%, 32.4%, 24.9%, and 9.3%, respectively. In terms of the most serious adverse events that occurred, the incidence of grade 1, 2, 3, and 4 were 38.2%, 32.4%, 9.8%, and 2.2%, respectively.

### Changes of the inflammatory and nutritional markers before and after NACT

According to the laboratory tests, the hemoglobin (Hgb), neutrophil count (Neut), and platelet count (Plt) significantly decreased after NACT (all *P* < 0.001), whereas no significant reduction was observed in the lymphocyte count (Lym) (*P* = 0.308). Oppositely, the monocyte (Mon) count (× 10^9^/L) significantly increased after NACT (0.37 ± 0.12 vs. 0.39 ± 0.13; *P* = 0.011) (Table 2).

**Table 2:** The comparisons of laboratory investigations, inflammatory and nutritional markers between pre-NACT and post-NACT

Markers	Pre-NACT	Post-NACT	*P*
Laboratory investigations			
Hgb (g/L)	130.8 (± 23.8)	126.4 (± 21.8)	**< 0.001**
Neut (× 10^9^/L)	3.80 (± 1.50)	3.08 (± 1.17)	**< 0.001**
Lym (× 10^9^/L)	1.67 (± 0.55)	1.64 (± 0.53)	0.308
Mon (× 10^9^/L)	0.37 (± 0.12)	0.39 (± 0.13)	**0.011**
Plt (× 10^9^/L)	249.2 (± 81.7)	196.5 (± 62.8)	**< 0.001**
Inflammatory markers			
NLR	2.57 (± 1.64)	2.08 (± 1.23)	**< 0.001**
PLR	165.35 (± 81.32)	129.46 (± 51.72)	**< 0.001**
LMR	4.87 (± 1.84)	4.52 (± 1.68)	**0.002**
SII	652.79 (± 517.65)	404.00 (± 239.48)	**< 0.001**
CRP^a^ (mg/L)	3.00 (± 7.97)	3.83 (± 10.71)	0.471
CAR^a^	0.074 (± 0.205)	0.088 (± 0.230)	0.616
mGPS^a^			1.000
0	126 (94.0)	126 (94.0)	
1	8 (6.0)	8 (6.0)	
PI^a^			1.000
0	125 (93.3)	126 (94.0)	
1	9 (6.7)	8 (6.0)	
Nutritional markers			
Alb (g/L)	42.65 (± 3.92)	42.64 (± 3.80)	0.963
BMI (kg/m^2^)	23.39 (± 3.40)	23.71 (± 3.21)	**< 0.001**
PNI	51.01 (± 5.16)	50.83 (± 4.79)	0.570
mSIS			0.335
0	174 (77.3)	169 (75.1)	
1	30 (13.3)	35 (15.6)	
2	21 (9.3)	21 (9.3)	

*P* values are marked in bold if less than 0.05

Alb, albumin; BMI, body mass index; CAR, C-reactive protein–albumin ratio; CRP, C-reactive protein; GPS, glasgow prognostic score; Hgb, hemoglobin; LMR, lymphocyte-to-monocyte ratio; Lym, lymphocyte count; mGPS, modified glasgow prognostic score; Mon, monocyte count; mSIS, modified systemic inflammation score; NACT, neoadjuvant chemotherapy; Neut, neutrophil count; NLR, neutrophil-to-lymphocyte ratio; PI, prognostic index; PLR, platelet-to-lymphocyte ratio; Plt, platelet count; PNI, prognostic nutrition index; SII, systemic immune-inflammation index

^a^Data from 134 paired patients

All inflammatory markers derived from the complete blood count, including NLR, PLR, LMR, and SII, were significantly reduced (all *P* < 0.01) after NACT. The markers derived from CRP, including CAR, mGPS, PI, and the CRP itself, showed no significant difference between pre-NACT and post-NACT (all *P* > 0.05). For the nutritional markers, the PNI, Alb, and mSIS showed no significant difference after NACT. Exceptionally, the BMI increased slightly after NACT (*P* < 0.001) (Table 2).

### Prognostic value of the inflammatory and nutritional markers

When only pre-NACT markers analyzed, the pre-NACT NLR and SII were selected as the predictive factors by the univariate analysis (Table 3), however, the multivariate analysis shows that none of the pre-NACT markers were the independent prognostic factor for OS. When only post-NACT markers analyzed, the univariate and multivariate analysis showed the post-NACT Hgb [hazard ratio (HR) = 0.982, 95% confidence interval (CI): 0.968 ~ 0.997, *P* = 0.015] was the independent prognostic factor for post-NACT OS (Tables 3 and 4). Lastly, given the situation when both pre-NACT and post-NACT markers available, the pre-NACT NLR, post-NACT LMR, post-NACT Hgb, and the change value of LMR were selected for multivariate analysis, by which the post-NACT Hgb (HR = 0.984, 95% CI: 0.970 ~ 0.998, *P* = 0.025) and the change value of LMR (HR = 1.183, 95% CI: 1.011 ~ 1.385, *P* = 0.036) were revealed to be the independent prognostic factors for post-NACT OS (Tables 3 and 4).

**Table 3:** The univariate analysis of inflammatory and nutritional markers

Situation	Only pre-NACT markers			Only post-NACT markers			Both pre-NACT and post-NACT markers								
	Pre-NACT			Post-NACT			Pre-NACT			Post-NACT			Change value^b^		
Adjustment	Age, sex, location, clinical stage			Age, sex, location, post-NACT clinical stage			Age, sex, location, clinical stage								
Survival	OS			Post-NACT OS											
	HR	95% CI	*P*	HR	95% CI	*P*	HR	95% CI	*P*	HR	95% CI	*P*	HR	95% CI	*P*
NLR	1.128	0.973 ~ 1.308	**0.059**	1.008	0.782 ~ 1.264	0.994	1.127	0.972 ~ 1.308	**0.068**	1.011	0.803 ~ 1.272	0.927	0.893	0.489 ~ 1.630	0.711
PLR	1.002	0.998 ~ 1.005	0.343	1.003	0.997 ~ 1.009	0.330	1.001	0.998 ~ 1.005	0.387	1.003	0.997 ~ 1.010	0.273	0.874	0.459 ~ 1.661	0.680
LMR	0.936	0.801 ~ 1.094	0.408	1.141	0.966 ~ 1.347	0.121	0.934	0.800 ~ 1.091	0.391	1.162	0.980 ~ 1.378	**0.084**	1.202	1.029 ~ 1.405	**0.020**
SII	1.000	1.000 ~ 1.001	**0.078**	1.000	0.999 ~ 1.002	0.390	1.000	1.000 ~ 1.001	0.100	1.001	1.000 ~ 1.002	0.275	0.948	0.479 ~ 1.876	0.878
CRP^a^	0.961	0.854 ~ 1.080	0.497	0.991	0.951 ~ 1.032	0.658	0.962	0.858 ~ 1.079	0.513	0.991	0.950 ~ 1.033	0.672	1.012	0.983 ~ 1.041	0.430
CAR^a^	0.254	0.004 ~ 18.29	0.530	0.631	0.116 ~ 3.428	0.594	0.278	0.004 ~ 18.26	0.549	0.624	0.109 ~ 3.581	0.596	1.773	0.472 ~ 6.657	0.396
mGPS^a^															
0	Ref			Ref			Ref			Ref			NA	NA	NA
1	0.231	0.017 ~ 3.061	0.266	0.795	0.176 ~ 3.586	0.765	0.262	0.022 ~ 3.116	0.289	0.793	0.177 ~ 3.548	0.762	NA	NA	NA
PI^a^															
0	Ref			Ref			Ref			Ref			NA	NA	NA
1	0.692	0.121 ~ 3.958	0.679	0.654	0.147 ~ 2.907	0.577	0.727	0.132 ~ 4.006	0.714	0.646	0.146 ~ 2.854	0.564	NA	NA	NA
mSIS															
0	Ref			Ref			Ref			Ref			NA	NA	NA
1	0.819	0.332 ~ 2.021	0.665	0.900	0.409 ~ 1.982	0.794	0.789	0.320 ~ 1.948	0.608	0.986	0.451 ~ 2.158	0.972	NA	NA	NA
2	1.009	0.391 ~ 2.605	0.984	1.692	0.731 ~ 3.923	0.220	0.966	0.374 ~ 2.500	0.943	1.692	0.726 ~ 3.942	0.223	NA	NA	NA
Hgb	0.991	0.978 ~ 1.003	0.151	0.982	0.968 ~ 0.997	**0.015**	0.990	0.978 ~ 1.003	0.138	0.982	0.968 ~ 0.996	**0.014**	0.990	0.973 ~ 1.008	0.286
Alb	0.980	0.910 ~ 1.055	0.595	0.997	0.923 ~ 1.077	0.659	0.980	0.910 ~ 1.054	0.581	0.977	0.906 ~ 1.053	0.542	0.998	0.932 ~ 1.069	0.965
BMI	1.010	0.930 ~ 1.100	0.811	0.982	0.892 ~ 1.079	0.700	1.012	0.931 ~ 1.100	0.776	0.989	0.902 ~ 1.084	0.816	0.839	0.659 ~ 1.067	0.152
PNI	0.971	0.918 ~ 1.027	0.300	1.008	0.946 ~ 1.074	0.805	0.971	0.918 ~ 1.026	0.291	1.006	0.943 ~ 1.073	0.858	1.194	0.679 ~ 2.098	0.538

*P* values are marked in bold if less than 0.1

Alb, albumin; BMI, body mass index; CAR, C-reactive protein–albumin ratio; CI, confidence interval; CRP, C-reactive protein; Hgb, hemoglobin; HR, hazard ratio; LMR, lymphocyte-to-monocyte ratio; mGPS, modified glasgow prognostic score; mSIS, modified systemic inflammation score; NACT, neoadjuvant chemotherapy; NA, not applicable; NLR, neutrophil-to-lymphocyte ratio; OS, overall survival; PI, prognostic index; PLR, platelet-to-lymphocyte ratio; PNI, prognostic nutrition index; ref, reference; SII, systemic immune-inflammation index

^a^Data from 142 pre-NACT patients and 177 post-NACT patients

^b^Change value was calculated by subtracting the pre-NACT value from the post-NACT value

**Table 4:** The multivariate analysis of inflammatory and nutritional markers

	Multivariate			C-index	AIC
	HR	95% CI	*P*		
Only pre-NACT markers				0.675	496.2
Model: age + sex + location + clinical stage					
Pre-NACT NLR	1.176	1.008 ~ 1.348	0.059		
Pre-NACT SII			0.593		
Only post-NACT markers				0.692	488.0
Model: age + sex + location + post-NACT clinical stage					
Post-NACT Hgb	0.982	0.968 ~ 0.997	**0.015**		
Both pre-NACT and post-NACT markers				0.719	485.1
Model: age + sex + location + clinical stage					
Pre-NACT NLR			0.395		
Post-NACT LMR			0.369		
Post-NACT Hgb	0.984	0.970 ~ 0.998	**0.025**		
ΔLMR	1.183	1.011 ~ 1.385	**0.036**		
Control model: ypTNM stage				0.706	469.8
Control model: age + sex + location + TRG + ypTNM stage				0.738	473.3

*P* values are marked in bold if less than 0.05

AIC, akaike information criterion; CI, confidence interval; Hgb, hemoglobin; HR, hazard ratio; LMR, lymphocyte-to-monocyte ratio; NACT, neoadjuvant chemotherapy; NLR, neutrophil-to-lymphocyte ratio; ref, reference; SII, systemic immune-inflammation index; TRG, tumor regression grade

**Fig. 1:**
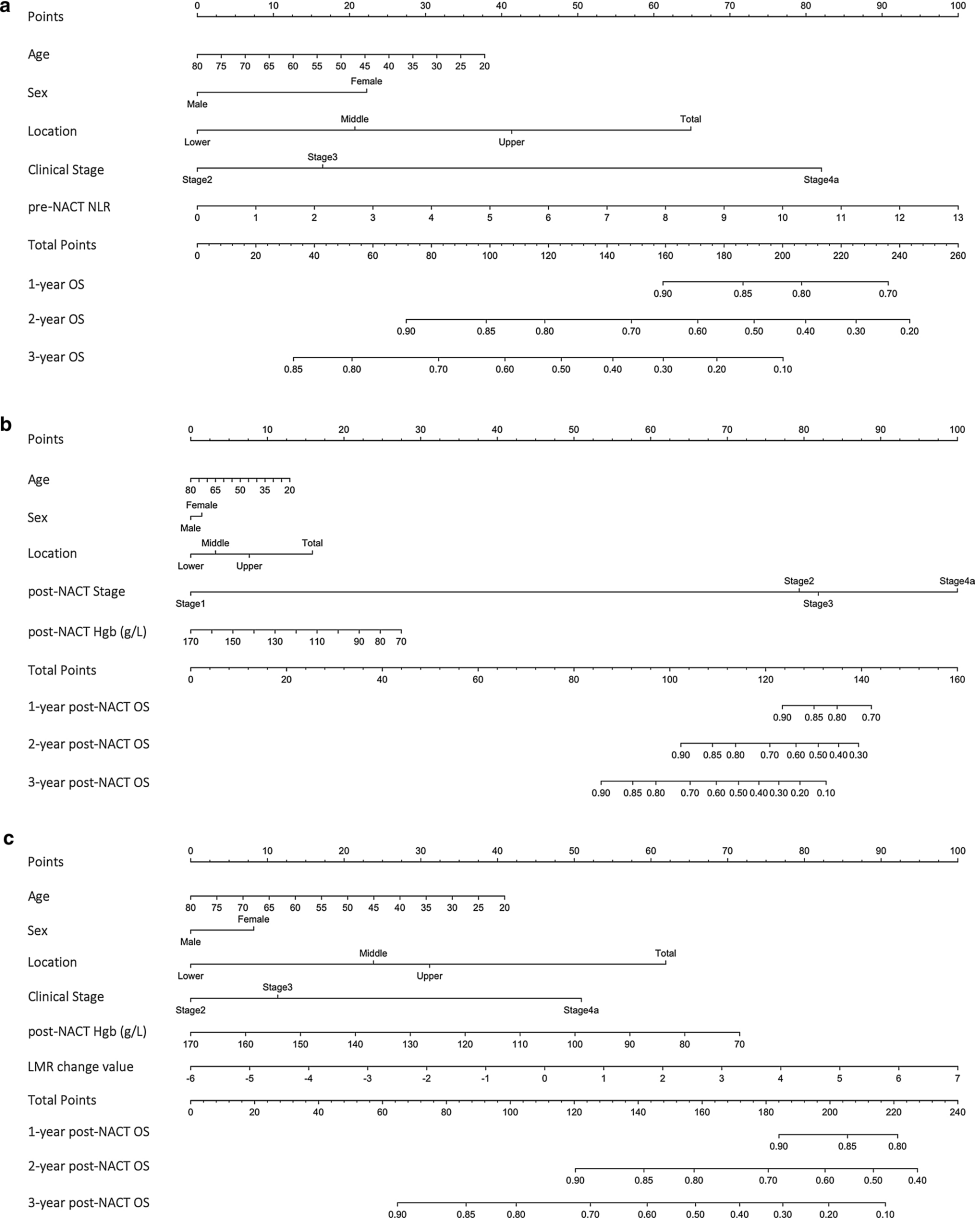
Nomogram predicting 1-year, 2-year, and 3-year survival for locally advanced gastric cancer with neoadjuvant chemotherapy and D2 lymphadenectomy. **a** When only pre-NACT markers are available, **b** when only post-NACT markers are available, **c** when both pre- and post-NACT markers are available, Hgb, hemoglobin; LMR, lymphocyte-to-monocyte ratio; NACT, neoadjuvant chemotherapy; OS, overall survival, change value was calculated by subtracting the pre-NACT value from the post-NACT value

The nomograms were plotted according to the hypothesized situation, using the corresponding models (Fig. 1). The C-index of the “only pre-NACT” model, “only post-NACT” model, and “both pre-NACT and post-NACT” model was 0.675, 0.692, and 0.719, respectively, which represented a moderate accuracy for survival prediction (Table 4). The ypTNM stage and the model including age, sex, tumor location, TRG, and ypTNM stage were used as the control model, of which the C-index was 0.706 and 0.738, respectively. In terms of the predictive accuracy, the model using both pre-NACT and post-NACT markers was slightly better than ypTNM stage alone (Table 4). However, in terms of the goodness of fitting, the ypTNM stage alone was the best among all the models (Table 4).

## Discussion

Most of the inflammatory and nutritional markers were derived from the easily accessible data and the commonly tested investigations; however, the clinical value and usage remained vague for patients with NACT. The pre-NACT and the post-NACT status might represent different clinical meanings, given the hypothesis that the patient’s inflammatory and nutritional status could be altered by NACT diversely. Thus, we collected both pre- and post-NACT data, and calculated most of the studied markers that were documented to be related to the prognosis for gastric cancer. All the markers analyzed were using the original values because of the lack of the unified classification criteria. We found that the levels of Hgb, Neut, and Plt were decreased, and the Mon count was increased significantly after NACT, but no significant differences presented in Lym levels. Accordingly, the changes subsequently led to the decrease of the post-NACT NLR, PLR, LMR, and SII, which might reflect the co-effects of hematocytopenia induced by NACT and the inflammation downregulation induced by tumor regression. Contrary to the inflammatory markers, most of the nutritional markers did not change significantly, except for BMI, which increased slightly but significantly after NACT. The presence of the alteration between pre-and post-NACT markers implied that the clinical value of these markers might be complicated depending on the time phases during the NACT.

In the three hypothesized clinical scenarios, the pre-NACT NLR (HR = 1.176, 95% CI: 1.008 ~ 1.348, *P* = 0.059) demonstrated a trend to correlate with poor OS with a boundary significance at the time of pre-NACT phase. The result was similar with the studies [18, 19], in which the pretreatment NLR was divided into high- and low-level, and the Cox models were not adjusted by clinical parameters. On the contrary, all the Cox models in the present study were adjusted by the clinical parameters, including age, sex, tumor location, and the clinical stage, which could help in minimizing the positive interaction between the advanced tumor stage and the poor prognosis. That could partially explain that, after the adjustment for clinical stage, the correlation between pre-NACT NLR and OS did not reach to the statistical significance.

When only post-NACT markers are taken into account, the post-NACT Hgb (HR = 0.982, 95% CI: 0.968 ~ 0.997, *P* = 0.015) was shown to be the independent prognostic factor. Interestingly, none of the post-NACT inflammatory markers could predict the post-NACT OS, a similar result was reported previously [19], the author holds that the post-NACT NLR lost its usefulness due to the bone marrow suppression. The studies in gastric and esophageal cancer [20, 21] also demonstrated that the development of anemia during NACT was associated with poor prognosis. In the present study, we further proved a negative quantitative correlation between the post-NACT Hgb and the post-NACT OS.

When both pre- and post-NACT markers are available, the post-NACT Hgb (HR = 0.984, 95% CI: 0.970 ~ 0.998, *P* = 0.025) and the change value of LMR (HR = 1.183, 95% CI: 1.011 ~ 1.385, *P* = 0.036) were the independent prognostic factors. Differently, the LMR itself was reported to have a positive correlation with the good prognosis, meaning the greater LMR value predicting, the better the survival. However, our result indicated that the post-NACT LMR was significantly lower than the pre-NACT value, which could be attributed to the significant increase of Mon count after NACT. A greater change value of LMR (representing a poor prognosis in our study) implied a lower pre-NACT LMR or a higher post-NACT LMR. Until now, only few studies concerned the issues about how were the inflammatory markers changed, and what were the impacts of the changes on the prognosis. According to the study [22] related to rectal cancer, the treatment-induced leukopenia correlated with a favorable OS; however, when it came to Lym, there was contradictory result reported [23]. Another study [24] related to gastric cancer revealed that the NACT had different effects on Lym depending on the subgroups. All these results implied the complexity and profundity of the impacts on host immunity induced by NACT. As the first study that performed the extensive analysis on the post-NACT markers and their changes in gastric cancer, we hold that it would be an interesting and intricate issue which needs further investigations.

None of the nutritional markers showed significant predictive values in the present study by either univariate or multivariate analysis. The reason might be attributed to, as an observational cohort study, the intervention bias from the inequality in nutritional support. That is, the malnutrition might be corrected temporarily to meet the requirements for NACT and surgery, and the nutritional markers might not reflect the status of autonomic nutrition level.

The ypTNM stage was a novel system produced to fulfill the need for the accurate survival prediction for gastric cancer with NACT. The comparison of the model containing the inflammatory markers with the ypTNM stage helped to evaluate the clinical values for these markers. The model containing age, sex, tumor location, clinical stage, post-NACT Hgb, and the change value of LMR showed a higher C-index compared to the ypTNM stage. More importantly, the parameters required for the model could be obtained preoperatively, which made it possible to make a good prediction before surgery, and facilitate the decision making.

The present study has a number of limitations. First, patients included in the analysis might have different supportive treatments, chemotherapy regimens and cycles, which could affect their immune and nutritional status differently. Failing to incorporate this information could have biased the estimates of current study. Second, the current length of follow-up is relatively short and therefore long-term survival cannot be assessed. Third, the nomogram developed has not been validated internally or externally, which would cast doubts on its generalizability.

## Conclusion

For locally advanced gastric cancer, the NACT could significantly decrease the Hgb Neut Plt, and increase Mon, leading the decline of NLR, PLR, LMR, and SII. The inflammatory and nutritional markers based on CRP and Alb were not significantly changed after NACT. The pre-NACT NLR (when only pre-NACT markers available), the post-NACT Hgb (when only post-NACT markers available), the post-NACT Hgb and the change value of LMR (when both pre- and post-NACT markers available) had some values in survival prediction combined with age, sex, tumor location and the clinical stages under different clinical scenarios. The elevated initial NLR, the preoperative anemia and the greater change value of LMR implied a poor prognosis.

## Electronic supplementary material

Below is the link to the electronic supplementary material.

Supplementary material 1 (DOCX 16 kb)

Supplementary material 2 (DOCX 15 kb)
