# Supplementary material for: The clinical value and usage of inflammatory and nutritional markers in survival prediction for gastric cancer patients with neoadjuvant chemotherapy and D2 lymphadenectomy
Source: Gastric Cancer. 2020 Feb 18;23(3):540–9. doi: 10.1007/s10120-019-01027-6 (PMC7165147; doi:10.1007/s10120-019-01027-6)
Supplement: Supplementary file 1 — Supplementary material 1 (DOCX 16 kb) [file 10120_2019_1027_MOESM1_ESM.docx]

**Supplementary Table 1 The formula of the inflammatory and nutritional markers**

| **Markers** | **Formula** |
| --- | --- |
| NLR | Neut / Lym |
| PLR | Plt / Lym |
| LMR | Lym / Mon |
| SII | Neut × Plt / Lym |
| CAR | CRP (mg/L) / Alb (g/L) |
| GPS | grade 0: CRP≤10 mg/L and Alb≥35g/L |
|  | grade 1: CRP＞10 mg/L or Alb＜35g/L |
|  | grade 2: CRP＞10 mg/L and Alb＜35g/L |
| mGPS | grade 0: CRP≤10 mg/L |
|  | grade 1: CRP＞10 mg/L and Alb≥35g/L |
|  | grade 2: CRP＞10 mg/L and Alb＜35g/L |
| PI | grade 0: CRP≤10 mg/L and WBC≤11×10^9^/L |
|  | grade 1: CRP＞10 mg/L or WBC＞11×10^9^/L |
|  | grade 2: CRP＞10 mg/L and WBC＞11×10^9^/L |
| mSIS | grade 0: Alb≥40g/L |
|  | grade 1: Alb＜40g/L and LMR≥3.4 |
|  | grade 2: Alb＜40g/L and LMR＜3.4 |
| BMI | Weight (Kg) / Height (m)^2^ |
| PNI | Alb (g/L) + Lym (10^9^/L) × 5 |

Alb, Albumin; BMI, body mass index; CAR, C-reactive protein–albumin ratio; CRP, C-reactive protein; GPS, Glasgow Prognostic Score; LMR, lymphocyte-to-monocyte ratio; Lym, Lymphocyte count; mGPS, modified Glasgow Prognostic Score; Mon, Monocyte count; mSIS, modified Systemic Inflammation Score; Neut, Neutrophil count; NLR, neutrophil-to-lymphocyte ratio; PI, Prognostic Index; PLR, platelet-to-lymphocyte ratio; Plt, Platelet count; PNI, prognostic nutrition index; SII, systemic immune-inflammation index; WBC, white cell count
