# Supplementary material for: The clinical value and usage of inflammatory and nutritional markers in survival prediction for gastric cancer patients with neoadjuvant chemotherapy and D2 lymphadenectomy
Source: Gastric Cancer. 2020 Feb 18;23(3):540–9. doi: 10.1007/s10120-019-01027-6 (PMC7165147; doi:10.1007/s10120-019-01027-6)
Supplement: Supplementary file 2 — Supplementary material 2 (DOCX 15 kb) [file 10120_2019_1027_MOESM2_ESM.docx]

**Supplementary Table 1 The adverse events during neoadjuvant chemotherapy**

| **Adverse events and grades** | **Case number** |
| --- | --- |
| Leucopenia |  |
| 0 | 129 (57.3) |
| 1 | 70 (31.1) |
| 2 | 24 (10.7) |
| 3 | 2 (0.9) |
| Neutropenia |  |
| 0 | 99 (44.0) |
| 1 | 68 (30.2) |
| 2 | 43 (19.1) |
| 3 | 13 (5.8) |
| 4 | 2 (0.9) |
| Thrombocytopenia |  |
| 0 | 152 (67.6) |
| 1 | 38 (16.9) |
| 2 | 25 (11.1) |
| 3 | 7 (3.1) |
| 4 | 3 (1.3) |
| Lymphocytopenia |  |
| 0 | 169 (75.1) |
| 1 | 38 (16.9) |
| 2 | 14 (6.2) |
| 3 | 4 (1.8) |
| Anemia |  |
| 0 | 130 (57.8) |
| 1 | 73 (32.4) |
| 2 | 19 (8.4) |
| 3 | 3 (1.3) |
| Hypoalbuminemia |  |
| 0 | 204 (90.7) |
| 1 | 15 (6.7) |
| 2 | 6 (2.7) |
| The grade of the most serious adverse events | |
| 0 | 39 (17.3) |
| 1 | 86 (38.2) |
| 2 | 73 (32.4) |
| 3 | 22 (9.8) |
| 4 | 5 (2.2) |

All adverse events were classified by the Common Terminology Criteria for Adverse Events
